# More than you can see: Unraveling the ecology and biodiversity of lichenized fungi associated with leaves and needles of 12 temperate tree species using high-throughput sequencing

**DOI:** 10.3389/fmicb.2022.907531

**Published:** 2022-09-16

**Authors:** Benjawan Tanunchai, Simon Andreas Schroeter, Li Ji, Sara Fareed Mohamed Wahdan, Shakhawat Hossen, Ann-Sophie Lehnert, Hagen Grünberg, Gerd Gleixner, François Buscot, Ernst-Detlef Schulze, Matthias Noll, Witoon Purahong

**Affiliations:** ^1^Department of Soil Ecology, UFZ-Helmholtz Centre for Environmental Research, Halle (Saale), Germany; ^2^Bayreuth Center of Ecology and Environmental Research, University of Bayreuth, Bayreuth, Germany; ^3^Biogeochemical Processes Department, Max Planck Institute for Biogeochemistry, Jena, Germany; ^4^School of Forestry, Central South of Forestry and Technology, Changsha, China; ^5^Department of Botany and Microbiology, Faculty of Science, Suez Canal University, Ismailia, Egypt; ^6^Institute of Bioanalysis, Coburg University of Applied Sciences and Arts, Coburg, Germany; ^7^Preßwitzer Straße, Unterwellenborn, Germany; ^8^German Centre for Integrative Biodiversity Research (iDiv) Halle-Jena-Leipzig, Leipzig, Germany

**Keywords:** ITS2, *Physcia adscendens*, red-list lichenized fungi, foliicolous lichens, Illumina MiSeq

## Abstract

Currently, lichen surveys are generally based on the examination of fruiting bodies. Lichens in the mycelial stage, in spores, or awaiting conditions for fruiting body formation are usually overlooked, even though they are important for maintaining biodiversity and ecosystem functions. This study aimed to explore the lichenized fungal community composition and richness associated with leaves and needles of 12 temperate tree species using Illumina MiSeq-based amplicon sequencing of the internal transcribed spacer (ITS) 2 region. *Picea abies* harbored the highest richness and number of lichenized fungal species. We found that the lichenized fungus *Physcia adscendens* dominated the leaves and needles of the most temperate tree species. Eleven lichenized fungal species detected in this study were recorded for the first time on leaves and needles. In addition, we identified *Athallia cerinella, Fellhanera bouteillei*, and *Melanohalea exasperata* that are on the German national red lists. Lichenized fungal richness was higher in conifer compared to broadleaf trees. Overall, tree species (within coniferous trees) and tree types (broadleaved vs. coniferous trees) harbored significantly different lichenized fungal community compositions pointing out the importance of host species. Diversity and community composition patterns of lichenized fungi were correlated mainly with tree species. Our study demonstrates that the diversity of foliicolous lichens associated with leaves and needles of 12 temperate tree species can be appropriately analyzed and functionally assigned using the ITS-based high-throughput sequencing. We highlighted the importance of conifers for maintaining the biodiversity of foliicolous lichens. Based on the discovery of many red list lichens, our methodological approach and results are important contributions to subsequent actions in the bio-conversation approaches.

## Introduction

Lichens are widely distributed in terrestrial ecosystems contributing to ecosystem functions such as biomass production and nutrient cycling, including carbon (C), nitrogen (N), and phosphorous (P) cycling (Plitt, 1919; Knops et al., 1996; Belnap et al., 2003; Asplund and Wardle, 2017). They are rich in nutrients (such as N and P) and, thus, significantly contribute to mineral cycling (Knops et al., 1996; Grimm et al., 2021). The occurrence and status of many lichens have been used for a long time as a bio-indicator for environmental air pollution (SO_2_), such pollution decreases photosynthesis and respiration rate in lichens (Beekley and Hoffman, 1981). Some other lichens have been proposed as passive monitors for climate change as their occurrence corresponds to the amount of humidity in the air (Lücking et al., 2009). Lichen diversity has been used to track the drivers of global change (e.g., temperature) as its diversity responds to the temperature (Matos et al., 2017). Lichens occupy a long-lived ecological niche in which symbiotic interactions occur with various lichen-associated microorganisms (mainly fungus, green algae, and/or cyanobacterium) (Grimm et al., 2021). Nowadays, lichens have been known to further host prokaryotes, lichenicolous fungi, and viruses (Cardinale et al., 2006; Petrzik et al., 2019; Grimm et al., 2021). A recent study revealed a complete carbon (C) cycling in a lichen symbiotic interaction between algal, fungal, and lichen partners (ten Veldhuis et al., 2020). The mycobiont shapes the thallus structure of lichens that allows better nutrient accessibility, whereas the photobiont (green algae) trades off C in form of sugar and O_2_ produced by photosynthesis to other microorganisms in the lichen symbiosis (Pinokiyo et al., 2006; ten Veldhuis et al., 2020; Grimm et al., 2021). Furthermore, the respiratory CO_2_ generated by the mycobiont can also be used by the photobiont (ten Veldhuis et al., 2020). The cyanobiont (a cyanobacterium) may be involved in both N_2_-fixation and photosynthesis as part of the symbiotic interactome (Grimm et al., 2021).

Lichen thalli play important roles both to secure the adherence of lichens onto different substrata and to embed and expose the photobiont to solar radiation (Grube and Hawksworth, 2007). Common lichen thallus forms are crustose (crust-like) and foliose (leaf-like) forms (Grube and Hawksworth, 2007; Grimm et al., 2021). Lichens that epiphytically colonize on live leaves/needles are so-called foliicolous lichens (Anthony et al., 2002; Pinokiyo et al., 2006). According to a previous study (Lücking et al., 2009), foliicolous lichens were re-classified into four groups: (i) truly foliicolous, (ii) ubiquitous, (iii) facultatively foliicolous, and (iv) accidentally foliicolous lichens. The differences among these foliicolous lichens are the variety of their substrates as outlined earlier (Lücking et al., 2009). Briefly, truly foliicolous lichens develop mainly on leaves and needles, while ubiquitous lichens can grow on various substrata, including leaves and needles, bark, rocks, and soils (Triebel et al., 2007; Lücking et al., 2009). Facultatively foliicolous lichens can change their lifestyle to grow on leaves or needles under certain limitations or environmental conditions (Lücking et al., 2009). These first three foliicolous lichen groups are considered truly foliicolous lichens as they start their development and complete their life cycles on leaves/needles, while the accidentally foliicolous lichens (iv) start growing from adjacent branches and overgrow evergreen needles (Lücking et al., 2009).

Leaves and needles of different tree species differed in various traits including nutrition, the persistence of leaves and needles onto the trees [deciduous (shedding) vs. evergreen (non-shedding)], specific leaf area, shapes, pH, and water content. The persistence of leaves and needles plays an important role in the presence of foliicolous lichens as they need at least 2–3 years to develop thalli with reproductive organs to achieve their life cycle (Lücking and Bernecker-Lücking, 2002). There is still controversial discussion on the nutrient uptake of foliicolous lichens from host leaves and needles (Anthony et al., 2002). Some believed that lichens can access the nutrients of their host leaves and needles, whereas some believed that lichens only epiphytically live there without using the nutrient from the hosts, but absorb light and shade their host leaves (Anthony et al., 2002). While the nutrient uptake of lichens from the host leaf is in debate, other environmental factors, such as leaf water content and pH, were reported to be important factors controlling lichenized fungi (Spier et al., 2010; Potkay et al., 2020). Lichens are poikilohydric organisms, they passively receive water from their substrates and environment (e.g., air humidity) (Lange et al., 1968; Grimm et al., 2021). The substrate preference, occurrence, and activity of lichens directly correspond with air humidity and moisture dynamics (Lücking et al., 2009; Potkay et al., 2020).

High-throughput or Next-Generation Sequencing approaches have revolutionized lichen biodiversity research (Grimm et al., 2021). The sequencing results, however, largely depend on the choice of barcode locus. Banchi et al. (2018) proposed the use of the internal transcribed spacer 2 (ITS2) gene to access the taxonomy and diversity of lichen mycobiome. They successfully detected and identified diverse lichenized fungal operational taxonomic units (OTUs). The fungal ITS gene is one of the most commonly targeted regions for detecting fungi, especially for the environmental sequencing substrates, such as leaf, soil, and wood. More than 2.6 million ITS sequences have been deposited in the UNITE database and the International Nucleotide Sequence Databases (Karsch-Mizrachi et al., 2018; Nilsson et al., 2019) (last accessed 28.03.2022). A large number of available sequences offers better accessibility for the molecular identification of fungi (Nilsson et al., 2009, 2019). UNITE database is a recently published web-based database containing more than one million public fungal ITS sequences for reference (Nilsson et al., 2019). These ITS sequences are clustered into UNITE species hypotheses that enable us to consistently identify and compare microbes from different datasets obtained from independent molecular techniques at the species level (Nilsson et al., 2019). This is the first time that the lichenized fungal diversity and community composition associated with leaves and needles of the 12 common temperate tree species are characterized using Illumina MiSeq and the most recent bioinformatic pipeline, Divisive Amplicon Denoising Algorithm 2 (DADA2) (Callahan et al., 2016). Lichens and their diversity have been used as important biological indicators for monitoring environmental quality (Abas, 2021). Studying lichenized fungal diversity and community composition is a crucial step in biodiversity conservation (Purahong et al., 2019).

This current study aims to (i) investigate the diversity and community composition of lichenized fungi associated with leaves and needles of 12 temperate tree species using high-throughput sequencing technique (Illumina MiSeq) based amplicon sequencing of the ITS2 region and the most recent bioinformatic pipeline (based on DADA2) in combination with the UNITE species hypothesis, and (ii) identify the corresponding factors shaping the diversity and community composition patterns of lichenized fungi. These factors include tree species, tree type, water content, pH, and location. We hypothesize that (i) our state-of-the-art sequencing approach is able to obtain overlooked lichenized fungi associated with leaves/needles of 12 temperate tree species at fine taxonomic levels, such as genus or species levels; and (ii) tree species and tree types are the significant factors shaping the diversity and community composition pattern of lichenized fungi associated with leaves/needles of 12 temperate tree species.

## Materials and methods

### Study site and sampling

The sampling site was located in the Hainich-Dün region of Thuringia, Germany (51°12'N 10°18'E, elevations ranging from 100 to 494 m above sea level) (Supplementary Figure S1). The characteristics of the study site are 600–800 mm mean annual precipitations and 6–7.5°C mean annual temperatures. The main soil type is a Cambisol on limestone as bedrock with the soil pH of 5.1. In total, a minimum of 200 g of healthy-looking leaves and needles were collected from branches at the lower part of the crown of the 60 mature trees (3-years-old leaves and needles, 12 temperate tree species, and 5 replicate tree individuals per species) in October 2019. The 12 temperate tree species consisted of eight broadleaved (including *Acer pseudoplatanus, Carpinus betulus, Fagus sylvatica, Fraxinus excelsior, Populus hybrid, Prunus avium, Quercus robur*, and *Tilia cordata*) and four coniferous tree species (including *Picea abies, Larix decidua, Pinus sylvestris*, and *Pseudotsuga menziesii*). *Fagus sylvatica* and *Picea abies* are the dominant tree species in this site. Leaves and needles of an individual tree were sampled in a separate sterilized plastic bag using new clean gloves. One set of samples was sent on ice to the physicochemical laboratory for water content and pH determination and another set of samples was transported on ice to the molecular laboratory within 3 h and stored at −80°C until further processing.

### Water content and pH measurement

To measure water content and leaf pH, wet leaf and needle samples were shaken for 1 h in falcon tubes with 30 mL Milli-Q water. The leachates were centrifuged for 5 min at 5,000 × *g*, decanted, and filtered through pre-flushed 0.45 μm regenerated cellulose syringe filters. The remaining leaf/needle material was dried for 2 weeks at 40°C for dry weight determination. The pH of the leachates was determined using pH paper with a scale precision of 0.2 pH units.

### DNA extraction and Illumina sequencing

DNA extraction and Illumina sequencing were done according to Tanunchai et al. (2022). In our previous study (Tanunchai et al., 2022), we compared FUNGuild and FungalTraits to assign fungal ASVs to lichenized fungi; however; we did not tackle the ecological aspect of those datasets. Briefly, for the preparation for the DNA extraction, up to 10 healthy-looking leaves and needles were carefully selected from five branches per tree individual to avoid contamination from the fungi living on branches. Loosely attached fungal mycelium and dust particles were removed from the leaf and needle sample by three-times washing with sterile Tween solution (0.1% vol/vol), then three to five times with MiliQ sterile water or until no bobble, and finally, incubated for 1 h in sterile water at room temperature. The samples were then ground using liquid N_2_ and sterilized nails, homogenized, then stored at −20°C for further analysis. The total fungal community attached firmly to the leaf and needle samples (~120 mg homogenized leaves and needles) was then subjected to DNA extraction using the DNeasy PowerSoil kit (Qiagen, Hilden, Germany) and a Precellys 24 tissue homogenizer (Bertin Instruments, Montigny-le-Bretonneux, France) according to the manufacturer's instructions. The total fungi were characterized by the fungal internal transcribed spacer (ITS)-based amplicon sequencing on the Illumina MiSeq sequencing platform, as outlined earlier (Weißbecker et al., 2020). The fungal ITS2 gene was amplified using the fungal primer pair fITS7 [5′- GTCTCGTGGGCTCGGAGATGTGTATAAGAGACAGNNNGTGARTCATCGAATCTTTG-3′ and 5′-GTCTCGTGGGCTCGGAGATGTGTATAAGAGACAGNNNNGTGARTCATCGAATCTTTG-3′] (Ihrmark et al., 2012) and ITS4 primer [5′-TCGTCGGCAGCGTCAGATGTGTATAAGAGACAGNNNNNTCCTCCGCTTATTGATATGC-3′ and 5′-TCGTCGGCAGCGTCAGATGTGTATAAGAGACAGNNNNNNTCCTCCGCTTATTGATATGC-3′] (White et al., 1990) with Illumina adapter sequences. Amplifications were performed using 20 μL reaction volumes with 5 × HOT FIRE Pol Blend Master Mix (Solis BioDyne, Tartu, Estonia). The amplified products were visualized by gel electrophoresis (fragment size length ~400 bp). Negative control was sequenced along with the samples. Paired-end sequencing (2 × 300 bp) was performed on the pooled PCR products (three technical replicates, ~45 μL reaction volumes) using a MiSeq Reagent kit v3 on an Illumina MiSeq system (Illumina Inc., San Diego, CA, United States) at the Department of Soil Ecology, Helmholtz Centre for Environmental Research, Germany as outlined earlier (Tanunchai et al., 2022).

### Bioinformatics

Bioinformatics analysis was conducted according to Tanunchai et al. (2022). Briefly, the ITS rRNA gene paired-end sequences were quality-trimmed from the demultiplexed raw reads using cutadapt (Martin, 2011), filtered for chimeras, and merged using the DADA2 package (Callahan et al., 2016) through the pipeline dadasnake (Weißbecker et al., 2020). Assembled reads fulfilling the following criteria were retained for further analyses: a minimum length of 70 nt, quality scores at least equal to 9 nt with a maximum expected error score of 5 nt for forward and reverse sequences, and no ambiguous nt. Merging was conducted with two mismatches allowed and a minimum overlap of 20 nucleotides required for fungal sequences. Fungal ASVs were classified against the UNITE v7.2 database (Kõljalg et al., 2013). A set of ASVs was classified using the Bayesian classifier as implemented in the mothur classify.seqs command, with a cut-off of 60 (Wang et al., 2007). DADA2 offers an advantage against the conventional OTU method as it results in less spurious sequences, which consequently reduces the inflation of the microbial richness. The inflation of the microbial richness is considered an important problem for OTU-based analyses (Callahan et al., 2016). The fungal raw reads were rarified. A total of 2,451 rarefied fungal ASVs with the minimum sequencing depths of 21,967 sequences per sample were obtained. The fungal ecological function of each ASV was determined using FUNGuild (Nguyen et al., 2016) and FungalTraits (Põlme et al., 2020) according to the authors' instructions. We assigned 59 lichenized fungal ASVs (accounting for 2.4% of total fungal ASVs and 0.87% of total fungal sequencing reads) by FUNGuild and FungalTraits (removing one fungal ASV with ambiguous ecological functions, *Sphaerulina*) (Tanunchai et al., 2022). Species of 59 lichenized fungal ASVs were identified by UNITE database based on UNITE species hypothesis. Lichenized fungal ASVs with identical UNITE species hypothesis and SH code were merged into a single species. In total, 28 lichenized fungal species were obtained. Relative abundance and richness of lichenized fungal species were used for further analyses. LIAS (Triebel et al., 2007) and peer-reviewed publications (Lücking, 1999; Lücking and Cáceres, 2002; Pinokiyo et al., 2006; Lücking et al., 2009; Grube, 2010) were used to affiliate traits to the lichenized fungal ASVs, such as identify distribution, substrata, record on leaf, and thallus forms. To confirm the ITS-based sequencing identification of lichenized fungi, we performed the fruiting body survey on the same temperate trees and collected visible fruiting bodies for microscopic examination.

### Statistical analysis

The datasets were tested for normality using the Jarque-Bera JB test and for the equality of group variances using the *F*-test (for two datasets) and Levene's test (for more than two datasets). To test the effect of tree species on lichenized fungal community composition, data of tree species with more than three replicates were considered. Effects of tree species and tree types on lichenized fungal community composition were visualized with principle coordinate analysis (based on relative abundance data and the Bray-Curtis distance measure) and tested with one-way PERMANOVA (based on relative abundance data and the Bray-Curtis distance measure), over 999 permutations of each run. The correlation analyses between pH, leaf/needle water content, and lichenized fungal species richness were performed using Spearman' rank correlation (ρ). The statistical differences in ASV richness among different tree species were performed using one-way ANOVA. All statistical analyses were performed using PAST version 2.17 (Hammer et al., 2001), R and RStudio version 4.0.4 (RStudio Team, 2019).

Specialist/generalist classification of the taxonomic dataset in this study was performed using the “EcolUtils” package in R and RStudio version 4.0.4 based on niche width and permutation algorithms. The indicator species were identified using the “indicspecies” package in R and RStudio version 4.0.4 based on the association between species patterns and combinations of groups of sites. The goodness-of-fit statistics (*R*^2^) of environmental variables fitted to the nonmetric multidimensional scaling (NMDS) ordination of lichenized fungal community composition were performed using the “Vegan” package in R and RStudio. The function “envfit” in the Vegan package fits environmental vectors or factors onto an ordination.

## Results

### Lichenized fungi associated with leaves and needles of 12 temperate tree species

**Figure 1:**
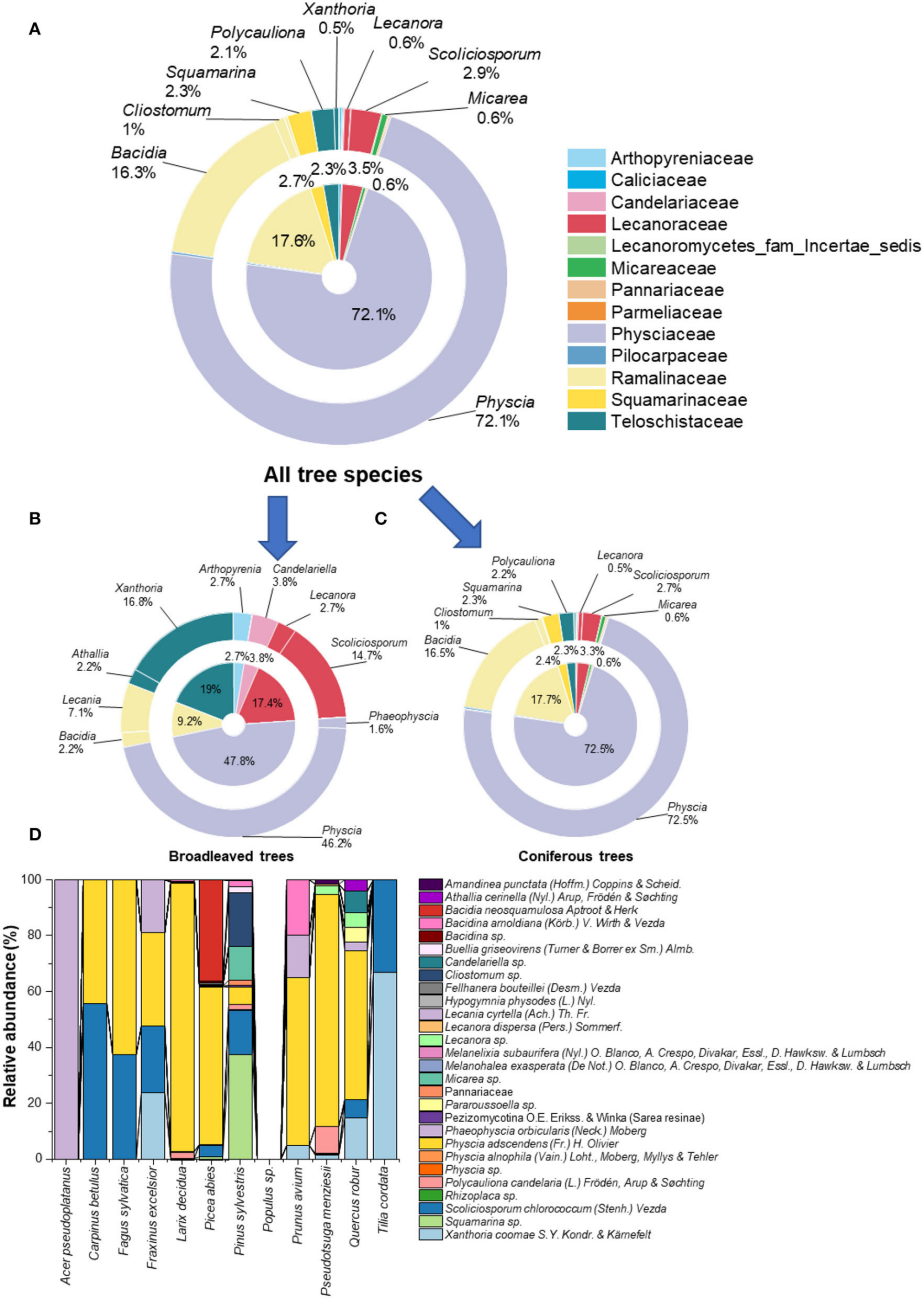
Proportion of lichenized fungi (at family- and genus level) associated with leaves and needles of all tree species **(A)**, leaves of broadleaved tree species **(B)**, needles of coniferous tree species **(C)**, and proportion of lichenized fungi (at species-level) **(D)** associated with leaves and needles of 12 temperate tree species (*n* = 5 tree replicates). No lichenized fungi were detected in *Populus* sp. (*Populus hybrid*). Color codes in **(A–C)** refer to lichenized fungal family given in the top-right of the figure.

Twenty-eight lichenized fungal species from 23 different genera were obtained (Figure 1; Table 1). In total, we assigned 11,508 fungal reads to lichenized fungal species (accounting for 0.87% of total fungal sequencing reads). Lichenized fungal reads of 11,324 (98%) were obtained from coniferous trees, while only 184 reads (2%) were obtained from broadleaved trees. The lichenized fungal genus, *Physcia* (accounting for 72.1% relative sequence read abundance of the total lichenized fungal community composition) dominated the lichenized fungal community composition across temperate tree species (including all coniferous tree and broadleaved trees; except the tree species maple, linden, and poplar), followed by lichenized fungal genera *Bacidia* (16.3%) and *Scoliciosporum* (2.9%) (Figure 1A). Similar patterns were found for lichenized fungal community composition associated with needles of coniferous trees, *Physcia* (72.5%), *Bacidia* (16.5%), and *Scoliciosporum* (2.7%) covered more than 90% relative abundance of the lichenized fungal community composition (Figure 1C). In broadleaved trees, *Physcia* (accounting for 46.2% relative sequence read abundance of the broadleaved associated lichenized fungal community composition) dominated the lichenized fungal community, followed by fungal genera, *Xanthoria* (16.8%), *Scoliciosporum* (14.7%), and *Lecania* (7.1%) (Figure 1B). The lichenized fungal genus *Physcia* is mainly represented by *Physcia adscendens* (Fr.) H. Olivier (Figure 1D). *Scoliciosporum chlorococcum* (Stenh.) Vezda co-dominated lichenized fungal community composition along with *Physcia adscendens* in three broadleaved tree species. In leaves of *Acer pseudoplatanus*, only *Phaeophyscia orbicularis* (Neck.) Moberg was detected and no lichenized fungi were detected in the leaves of the *Populus hybrid* (Figure 1D).

**Table 1:** UNITE species hypothesis of lichenized fungi associated with leaves and needles of 12 temperate tree species with the information on its global distribution, red-list status (recorded in Germany), indicator species, substrate, record on leaf/needle, and thallus form.

**Final UNITE species hypothesis (percent match > 97%)**	**Host tree**	**Indicator species for**	**Fruiting body survey**	**Sequence record in Germany**	**Red-list status (Germany)**	**Red-list status (Thuringia)**	**Global occurrence (LIAS)**	**Substrata (LIAS)**	**Record on leaf/needle**	**Thallus** **form (LIAS)**
*Amandinea punctata* (Hoffm.) Coppins and Scheid.	DG, KI	NA	NA	NA	Not threatened	Not threatened	Worldwide	Wood, bark, cork, plant surface, trunks, branches, twigs, rock	Yes	Crustose
*Athallia cerinella* (Nyl.) Arup, Frödén and Søchting	EI	NA	BU	NA	**Highly threatened**	NA	Australia, Europe, Americas	Wood, bark, cork, plant surface, trunks, branches, twigs	NA	Crustose
*Bacidia neosquamulosa* Aptroot and Herk	FI	NA	NA	NA	Not threatened	NA	Europe, Americas	Bark, cork, plant surface, trunks, branches, twigs	NA	Crustose, squamulose, subsquamulose
**(Specialist)**										
*Bacidina arnoldiana* (Körb.) V. Wirth and Vezda	KB, KI, LA	NA	NA	NA	Not threatened	**Threatened**	Asia, Europe, Americas	Rock	Yes	Crustose
									(accidentally foliicolous lichen)	
*Bacidina* sp.	FI	NA	NA	NA	NA	NA	NA	NA	NA	NA
*Buellia griseovirens* (Turner and Borrer ex Sm.) Almb.	KI	NA	BU	NA	Not threatened	Not threatened	Australia, Europe, Americas	Wood, bark, cork, plant surface, trunks, branches, twigs	NA	Crustose
*Candelariella* sp.	EI, FI, LA	NA	BU	NA	NA	NA	NA	NA	NA	NA
*Cliostomum* sp.	KI	NA	NA	NA	NA	NA	NA	NA	NA	NA
**(Specialist)**										
*Fellhanera bouteillei* (Desm.) Vezda	FI	NA	NA	NA	**Threatened with extinction**	Not threatened	Worldwide except Africa and Antarctica	Bark, cork, plant surface, trunks, branches, twigs, leaves, fronds, needles, rock	Yes (ubiquitous foliicolous lichen)	Leprose, crustose
*Hypogymnia physodes* (L.) Nyl.	FI, LA	NA	FI, LA	NA	Not threatened	Not threatened	Worldwide except Antarctica and Australia	Soil, humus, turf, detritus, dead leaves, mosses, wood, bark, cork, plant surface, trunks, branches, twigs, rock	Yes	Foliose
*Lecania cyrtella* (Ach.) Th. Fr.	AH, ES, FI, KB	FI	BU	NA	Not threatened	Not threatened	Worldwide except Antarctica and Australia	Bark, cork, plant surface, trunks, branches, twigs	NA	Crustose
*Lecanora dispersa* (Pers.) Sommerf.	DG	NA	NA	NA	Not threatened	NA	Worldwide except Antarctica	Soil, humus, turf, detritus, dead leaves, bark, cork, plant surface, trunks, branches, twigs, rock	Yes	Crustose
*Lecanora* sp.	Coniferous trees (except KI) and EI	NA	NA	NA	NA	NA	NA	NA	NA	NA
*Melanelixia subaurifera* (Nyl.) O. Blanco, A. Crespo, Divakar, Essl., D. Hawksw. and Lumbsch	FI	NA	FI, LA	NA	Not threatened	Not threatened	Asia, Europe, Americas	Mosses, wood, bark, cork, plant surface, trunks, branches, twigs, rock	NA	Foliose
*Melanohalea exasperata* (De Not.) O. Blanco, A. Crespo, Divakar, Essl., D. Hawksw. and Lumbsch	LA	NA	NA	NA	**Highly threatened**	**Threatened**	Worldwide except Antarctica and Australia	Wood, bark, cork, plant surface, trunks, branches, twigs, rock	NA	Foliose
*Micarea* sp.	KI	NA	NA	NA	NA	NA	NA	NA	NA	NA
**(Specialist)**										
Pannariaceae sp.	KI	NA	NA	Yes	NA	NA	NA	NA	NA	NA
*Pararoussoella* sp.	EI	NA	NA	Yes	NA	NA	NA	NA	NA	NA
Pezizomycotina O.E. Erikss. and Winka (*Sarea resinae*) *	KI	KI	NA	Yes	Near threatened	NA	Europe, Americas	Bark, cork, plant surface, trunks, branches, twigs	NA	Crustose
*Phaeophyscia orbicularis* (Neck.) Moberg	EI	NA	BU	Yes	Not threatened	Not threatened	Worldwide except Antarctica	Bark, cork, plant surface, trunks, branches, twigs, rock	NA	Foliose
*Physcia adscendens* (Fr.) H. Olivier **(Generalist)**	All coniferous tree and broadleaved trees (except AH, LI, PA)	DG, FI, and LA	BU, FI, LA	Yes	Not threatened	Not threatened	Worldwide	Wood, bark, cork, plant surface, trunks, branches, twigs, rock	Yes (accidentally foliicolous lichen)	Foliose
										
*Physcia alnophila* (Vain.) Loht., Moberg, Myllys and Tehler	LA	NA	LA	NA	NA	NA	Europe, Americas	NA	NA	Foliose
*Physcia* sp.	FI, LA	NA	NA	Yes	NA	NA	NA	NA	NA	NA
*Polycauliona candelaria* (L.) Frödén, Arup and Søchting	Coniferous trees (except FI)	DG and LA	NA	Yes	Not threatened	NA	Worldwide except Australia	Wood, bark, cork, plant surface, trunks, branches, twigs, rock	NA	Foliose, subfruticose
*Rhizoplaca* sp.	DG, KI	NA	NA	NA	NA	NA	NA	NA	NA	NA
*Scoliciosporum chlorococcum* (Stenh.) Vezda	Coniferous trees (except LA) and broadleaved trees (except AH, KB, PA)	FI and KI	NA	NA	Not threatened	**Vulnerable fungi**	Europe, Americas	Bark, cork, plant surface, trunks, branches, twigs	NA	Crustose
*Squamarina* sp.	Coniferous trees (except DG)	FI and KI	NA	NA	NA	NA	NA	NA	NA	NA
*Xanthoria come* S.Y. Kondr. and Kärnefelt	Coniferous trees (except KI) and EI, ES, KB, LI	DG, EI, and ES	BU, FI, LA	Yes	NA	NA	NA	Bark	NA	Foliose
**(Generalist)**										

*According to UNITE database, it was identified as *Sarea resinae* (Near threatened), however, the UNITE species hypothesis identified it as Pezizomycotina O.E. Erikss. and Winka. Thus, we exclude it from the red-list species.

Information about specialists and generalists is given with the species name. Host tree species abbreviations are: AH, *Acer pseudoplatanus*; BU, *Fagus sylvatica*; DG, *Pseudotsuga menziesii*; EI, *Quercus robur*; ES, *Fraxinus excelsior*; FI, *Picea abies*; HBU, *Carpinus betulus*; KB, *Prunus avium*; KI, *Pinus sylvestris*: LA, *Larix decidua*; LI, *Tilia cordata*; PA, *Populus hybrid*; NA, Not assigned. Information from the lichens and non-lichenized ascomycetes (LIAS) was taken from Triebel et al. (2007).

Bold letters indicate red-list status and generalist/specialist.

The lichenized fungal species detected in this study were classified into two main types of lichens according to their thallus form, (i) crustose (e.g., *Amandinea punctata, Fellhanera bouteillei*, and *Lecanora dispersa*) and (ii) foliose (e.g., *Hypogymnia physodes, Physcia adscendens*, and *Polycauliona candelaria*) (Table 1). Some lichens also had more than one thallus form, e.g., *Fellhanera bouteillei* (crustose and leprose) and *Polycauliona candelaria* (foliose and subfruticose). The foliicolous lichen, *F. bouteillei*, which was found on needles of young planted *Picea abies*, was recorded as red-list species with the status “threatened with extinction” according to the German national red lists (Wirth et al., 2011). In addition, two other lichenized fungal species with the red-list status of highly threatened (*Athallia cerinella* (Nyl.) Arup, Frödén and Søchting on leaves of *Quercus robur* and *Melanohalea exasperata* (De Not.) O. Blanco, A. Crespo, Divakar, Essl., D. Hawksw. and Lumbsch on needles of planted *Larix decidua*) were observed (Table 1). On the other hand, *Bacidina arnoldiana* (Körb.) V. Wirth and Vezda and *Melanohalea exasperata* were listed with the status “threatened” according to the red-list species of Thuringia (Eckstein and Grünberg, 2021). Moreover, we identified seven indicator species for coniferous tree species [including *Lecania cyrtella*, Pezizomycotina *(Sarea resinae), Polycauliona candelaria, Scoliciosporum chlorococcum, Squamarina* sp., *P. adscendens*, and *Xanthoria coomae*] (Table 1). Based on niche width and permutation algorithms, we assigned three lichenized fungal specialists (including *Bacidia neosquamulosa, Cliostomum* sp., and *Micarea* sp.) and two generalists (including *P. adscendens* and *X. coomae*) (Table 1).

### Link between next-generation sequencing and fruiting body data sets for species identification of lichenized fungi

To confirm the ITS-based sequencing identification of lichenized fungi, we collected the fruiting bodies for microscopic examination from the same temperate trees. There were 10 out of 28 lichenized fungal species detected by NGS that were also found by the fruiting body survey (Table 1). These lichens include *Athallia cerinella, Buellia griseovirens, Candelariella* sp., *Hypogymnia physodes, Lecania cyrtella, Melanelixia subaurifera, Phaeophyscia orbicularis, Physcia adscendens, P. alnophila*, and *X. coomae*. However, five lichenized species (*A. cerinella, B. griseovirens, Candelariella* sp., *L. cyrtella*, and *P. orbicularis*) detected by NGS were found on different host trees than those examined in the fruiting body survey.

### Richness of lichenized fungi associated with leaves and needles of 12 temperate tree species

**Figure 2:**
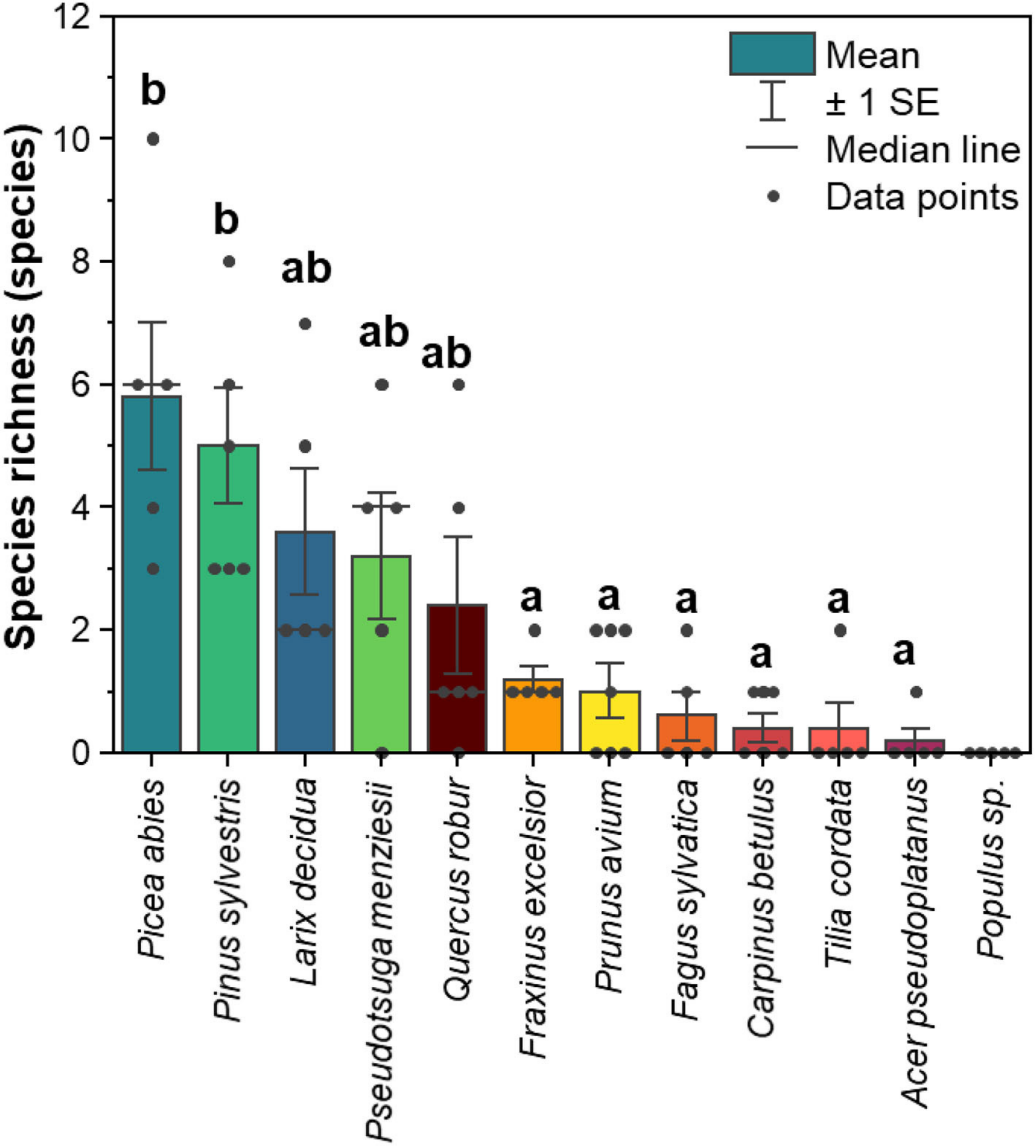
Mean of species richness of lichenized fungi associated with leaves and needles of 12 temperate tree species (*n* = 5 tree replicates). Yellow-red-brown color tone refers to the broadleaved tree species and blue-green color tone refers to the coniferous tree species. The statistical differences (*P* < 0.05) as indicated by letters between species richness among different tree species [excluding *Populus* sp. (*Populus hybrid*)] were performed using one-way ANOVA.

The richness of lichenized fungal species (merge of ASVs with identical UNITE species hypothesis) was higher in coniferous trees than in broadleaved trees (Figure 2). Among coniferous trees, *Picea abies* harbored the highest species richness (average of five replicate trees = 5.8 species, ranging from 3 to 10 species), while *Pseudotsuga menziesii* revealed the lowest species richness (average of five replicate trees = 3.2 species, ranged from 0 to 6 species). *Quercus robur* harbored the highest species richness (average of five replicate trees = 2.4 species, ranging from 0 to 6 species) among broadleaved trees, whereas, in *Populus hybrid*, no lichenized fungi were detected.

A similar pattern was found when we considered the total number of lichenized fungal species detected in each tree species. In total, conifers harbored 25 out of 28 lichenized fungal species. Among conifers, *Picea abies* harbored the highest number of lichenized fungal species (13 species), followed by *Pinus sylvestris* (12 species), *Larix decidua* (11 species), and *Pseudotsuga menziesii* (8 species). In total, broadleaved tree species harbored 10 out of 28 lichenized fungal species. Among broadleaved tree species, *Quercus robur* harbored the highest number of lichenized fungal species (eight species), followed by *Fraxinus excelsior* (four species), and *Prunus avium* (four species). *Carpinus betulus, Fagus sylvatica*, and *Tilia cordata* harbored two lichenized fungal species, and *Acer pseudoplatanus* harbored solely one lichenized fungal species [*Lecania cyrtella* (Ach.) Th. Fr.].

### Leaves and needles and environmental factors shaped lichenized fungal community composition

**Figure 3:**
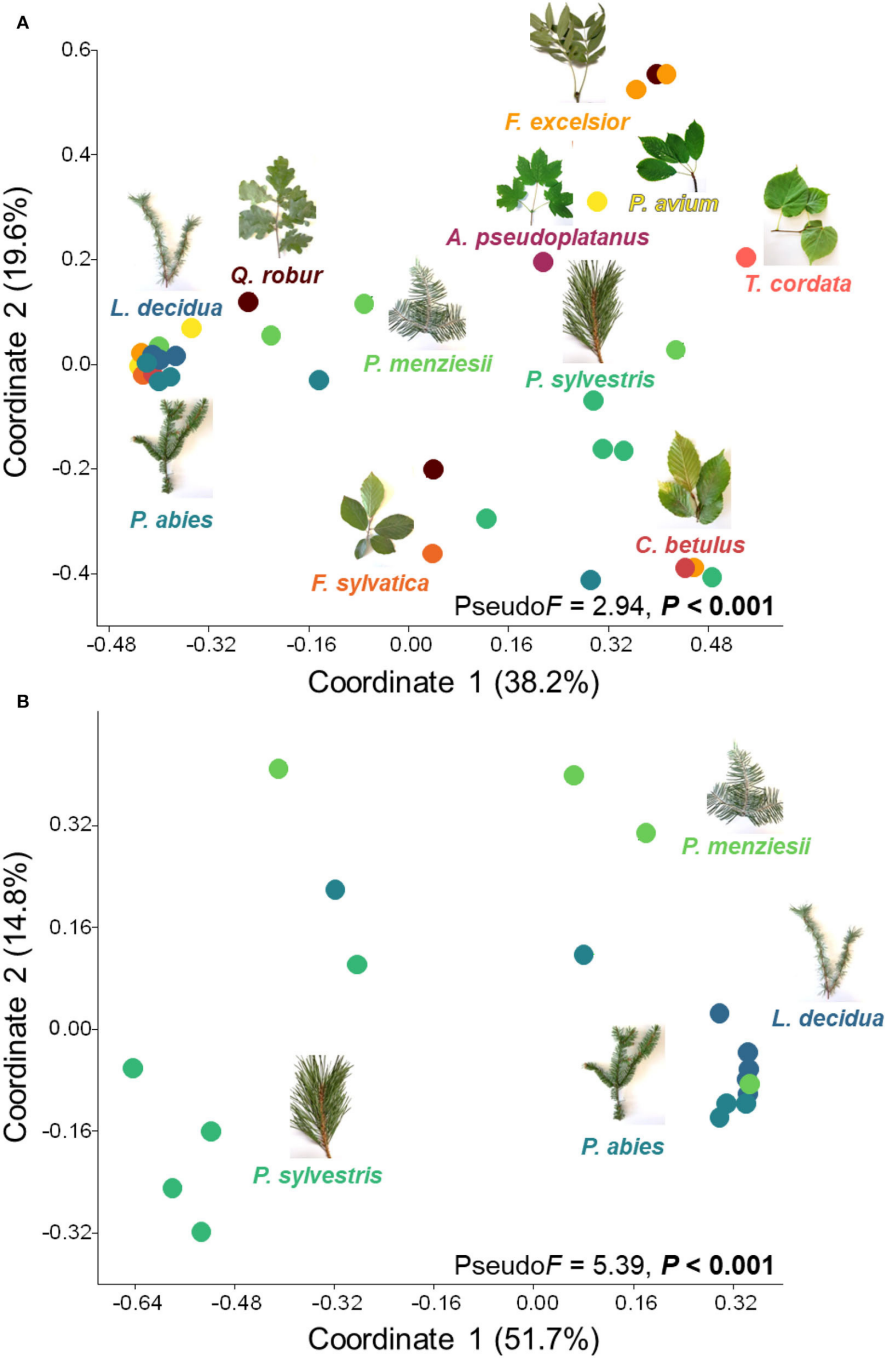
Principal coordinates showing lichenized fungal community compositions in all tree species **(A)** and coniferous tree species **(B)**. Effects of tree species (only tree species that contain lichenized fungi in more than three replicates are considered) were tested with one-way PERMANOVA (based on relative abundance data and the Bray-Curtis distance measure). *Populus hybrid*. was excluded from the analysis. Color code of each data point indicates leaves and needles of different tree species and is consistent with the color code of Figure 2.

Overall, the lichenized fungal community compositions significantly differed among 11 temperate tree species (Pseudo*F* = 2.94, *P* < 0.001; Figure 3A). The lichenized fungal community compositions associated with needles of *Pinus sylvestris* and *Larix decidua* were significantly different from those of almost all other tree species (Supplementary Table S1). Considering coniferous tree species, the lichenized fungal community compositions also significantly differed (Pseudo*F* = 5.39, *P* < 0.001; Figure 3B), except for *Picea abies* and *Pseudotsuga menziesii* (Supplementary Table S1). Considering broadleaved tree species (*Fraxinus excelsior, Prunus avium*, and *Quercus robur*), no significant difference was detected (Pseudo*F* = 0.93, *P* > 0.05; Supplementary Figure S2).

**Figure 4:**
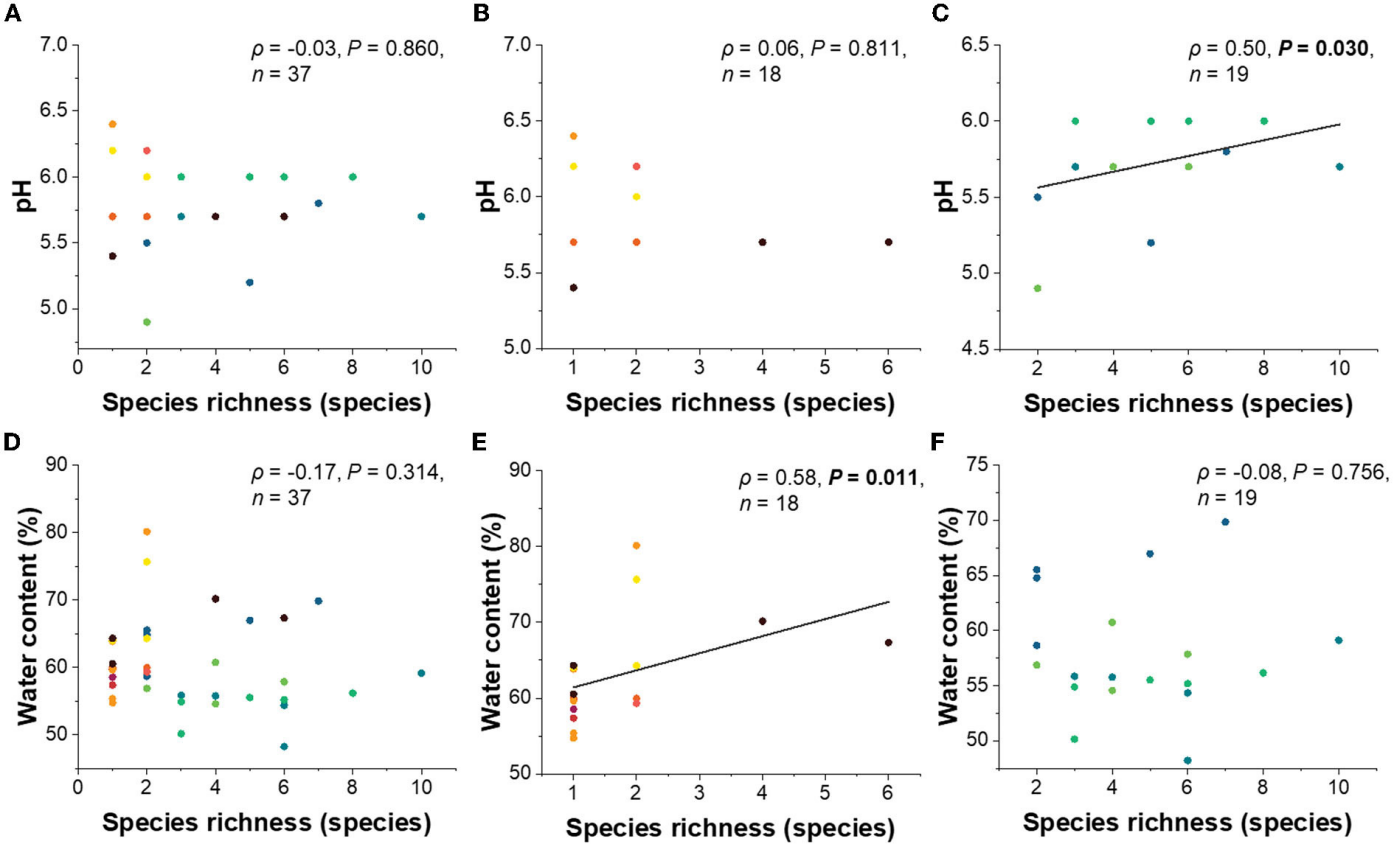
Linear regressions between lichenized fungal species richness of all tree species **(A)**, broadleaved tree species **(B)**, coniferous tree species **(C)**, and pH. Linear regressions between lichenized fungal species richness of all tree species **(D)**, broadleaved tree species **(E)**, coniferous tree species **(F)**, and water content. Significance was tested using Spearman' rank correlation (ρ). Color code of each data point indicates leaves and needles of different tree species and is consistent with the color code of Figure 2. one point in the figure may contain several data, because some tree species have the same pH value or water content and lichenized species richness.

The goodness of fit tests showed that tree species, tree type, and water content significantly corresponded with the lichenized fungal community composition (Table 2). When needles of coniferous trees were considered, tree species, pH, and longitude significantly corresponded with lichenized fungal community composition. The lichenized fungal richness of all tree species had no significant correlation with pH and water content (Figure 4). However, when considering broadleaved and conifer trees separately, pH (ρ = 0.50, *P* = 0.030) was found to be the important factor that significantly positively correlated with lichenized fungal species richness of conifer and water content positively correlated with those of broadleaf (ρ = 0.58, *P* = 0.011) (Figure 4).

**Table 2:** Goodness-of-fit statistics (*R*^2^) of environmental variables fitted to the nonmetric multidimensional scaling (NMDS) ordination of lichenized fungal community composition based on relative abundance data for three comparisons: (i) leaves and trees of all tree species, and separately for (ii) coniferous or (iii) broadleaved trees.

	**(i) All tree species**		**(ii) Coniferous trees**		**(iii) Broadleaved trees**	
	***R* ^2^**	***P***	***R* ^2^**	***P***	***R* ^2^**	***P***
Tree species	**0.53**	**0.001**	**0.67**	**0.001**	0.32	0.598
Tree type	**0.15**	**0.004**	NA	NA	NA	NA
pH	0.11	0.146	**0.37**	**0.031**	0.00	0.981
Water content	**0.25**	**0.008**	0.14	0.284	0.27	0.096
Latitude	0.13	0.105	0.19	0.190	0.10	0.491
Longitude	0.11	0.135	**0.36**	**0.023**	0.21	0.190

Bold letter indicates statistical significances. The data on leaf/needle water content and pH are provided in Supplementary Figure S3.

## Discussion

### The detection of lichenized fungi living on leaves and needles using high-throughput sequencing technique based on the amplicon sequencing of the ITS2 region

**Figure 5:**
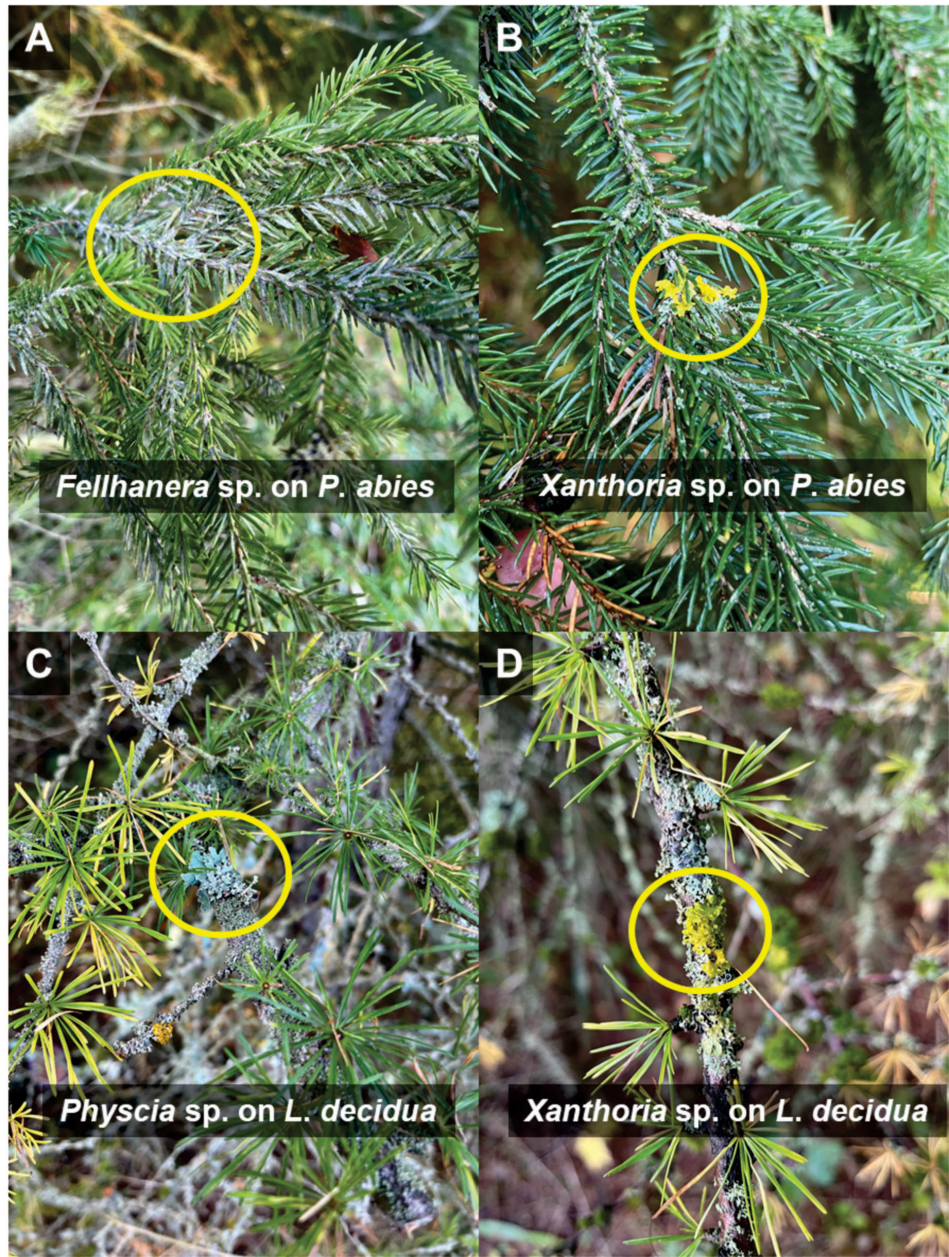
Lichens with visible fruiting bodies growing on branches and needles of *Picea abies* **(A,B)** and *Larix decidua* **(C,D)**. The identification of lichens was performed by Hagen Grünberg, Lichen-Expert Thüringen.

The observation of lichens by the experts was traditionally based on the presence and microscopic observations of fruiting bodies. In our study site, we observed lichens by eye on branches and needles of coniferous tree species, such as *Fellhanera, Physcia*, and *Xanthoria* (Figure 5). The contamination from lichens that grow on branches was unlikely as the leaf and needle samples were carefully selected, separated from branches, and washed three times with 0.1% sterile tween and five times with MiliQ sterile water. A high-resolution molecular technique based on the ITS2 region allowed the identification of the lichenized fungal taxonomy at fine taxonomic levels (genus or species level). This is consistent with a recent study (Banchi et al., 2018), which demonstrated that the fungal ITS2 gene can be efficiently used to access the taxonomy and diversity of lichen mycobiome. In fact, the fungal ITS gene is one of the most commonly targeted regions for detecting diverse groups of fungi, including saprotrophs, pathogens, endophytes, and lichenized fungi. Previous studies clearly reported the distinctiveness between lichenized fungi and fungal endophytes living in both lichen thalli and plants in terms of taxonomy, diversity, transmission mode, and evolutionary history (Arnold et al., 2009; U'Ren et al., 2010). In this study, we specifically select the lichenized fungal group for the analysis which is assigned function by the most successful and recently published annotation tools, FUNGuild (Nguyen et al., 2016) and FungalTraits (Põlme et al., 2020). We confirmed the lichenized fungal species using UNITE species hypothesis (Nilsson et al., 2019) and previously published literature, including LIAS (Triebel et al., 2007) and peer-reviewed publications (Lücking, 1999; Lücking and Cáceres, 2002; Pinokiyo et al., 2006; Lücking et al., 2009; Grube, 2010).

### Frequently overlooked high diversity of lichenized fungi were found on leaves and needles of 12 temperate tree species

More than 60% (17 out of 28 species) of lichenized fungal species were identified at the species level using the UNITE species hypothesis (Nilsson et al., 2019). Overall, we detected two times more diverse lichenized fungal species using NGS as compared to the traditional fruiting body survey (Table 1). Specifically, only 10 lichens were observed by a fruiting body survey and under the microscope. *P. adscendens* is one of the most widespread lichenized fungi detected in high relative abundance across both broadleaved (*Carpinus betulus, Fraxinus excelsior, Fagus sylvatica, Prunus avium*, and *Quercus robur*) and coniferous tree species (*Larix decidua, Picea abies, Pseudotsuga menziesii*, and *Pinus sylvestris*) in a temperate forest of Central Germany. *Physcia adscendens* is foliose lichen previously recorded worldwide (Triebel et al., 2007). It was classified as accidentally foliicolous lichen (Lücking et al., 2009) as it starts developing on branches and overgrown leaves. Accidentally foliicolous lichens are not considered truly foliicolous lichens, which would need at least 2 years to develop thalli with reproductive organs and complete their life cycle on leaves (Lücking and Bernecker-Lücking, 2002). Thus, the presence of *P. adscendens* on leaves and needles of deciduous trees, which shed their leaves or needles annually, may not be surprising.

In this study, needles of coniferous tree species harbor more rich and diverse lichenized fungal species as compared with leaves of broadleaved deciduous tree species. Specifically, needles of *Picea abies* harbored the highest richness and number of lichenized fungal species. Lichenized fungal community composition on needles of deciduous *Larix decidua* differs from other evergreen coniferous tree species. The high lichenized fungal species richness on needles of coniferous tree species may be explained by first, the persistence of evergreen needles which allows the development of lichens to achieve their life cycle (Lücking and Bernecker-Lücking, 2002). Second, fungal hyphae may attach better to the cuticular crust of needles. The main type of substrate or habitat, on which lichens were previously recorded, were bark, wood, and branches (Table 1) (Triebel et al., 2007). Interestingly, among the 17 lichenized fungal species, only six lichenized fungi were previously reported on leaves and only three lichenized fungi were classified as foliicolous lichens [*F. bouteillei* (Desm.) Vezda, *H. physodes*, and *Lecanora dispersa*] (Table 1). Other 11 lichenized fungi are recorded for the first time on leaf and needle substrata. These lichenized fungi included *A. cerinella, B. neosquamulosa, B. griseovirens, L. cyrtella, M. subaurifera, M. exasperata, P. orbicularis, P. alnophila, P. candelaria, S. chlorococcum*, and *X. coomae* (Table 1). These lichenized fungi are likely to be new potential accidentally foliicolous lichens. Furthermore, the Illumina MiSeq molecular technique is also able to capture red-list lichenized fungi, which are listed in the German national red lists with the status of “highly threatened” and “threatened with extinction” (Wirth et al., 2011). These include *A. cerinella, F. bouteillei*, and *M. exasperata*. To date, there is still no evidence of the free-living lichenized fungi (Tuovinen et al., 2015). The foliicolous or red-list lichenized fungi detected using the NGS could be in the life states of mycelium, in spores, or in the initial state to produce a fruiting body (Glassman et al., 2016; Purahong et al., 2022).

### Monitoring and conservation aspects of lichenized fungi using the high-throughput sequencing technique

The monitoring of lichens can be limited to those that have a fruiting body and can be observed under a microscope. However, overlooked or even invisible lichens living on leaves and needles may also play roles in the ecosystems such as indicator species for tree species and some are red-list lichenized fungal species. We found the importance of conifer needles as a habitat for foliicolous lichens as they harbored 98% of the lichenized fungal sequence reads and 89% of the lichenized fungal species (25/28 species) detected in this study. Thus, the maintenance of conifer trees is important for biodiversity conservation as they host a broad diversity of foliicolous lichens. This knowledge of lichen substrates is crucial to develop and apply targeted bio-conservation strategies (Purahong et al., 2019). Next-generation sequencing combined with the use of annotation tools (such as FungalTraits and FUNGuild) allows scientists without expert knowledge of lichens to identify this group of fungi and assign their functions. The sequencing database needs to be expanded to include verified DNA samples from described lichens so that we can get a more complete picture of invisible lichens. Our study is preliminary showing that lichens associated with leaves and needles are more diverse than so far expected by microscopic fruiting body surveys or examinations by the naked eye.

### Factors determining community composition patterns and richness of lichenized fungi

Tree species and tree type are known to determine the fungal community composition in deadwood of different tree species due to the different physicochemical properties (Bantle et al., 2014a,b; Purahong et al., 2018). Consistent with those observations, the majority of lichenized fungal community compositions are shaped by the leaves and needle substrate of the respective tree species and tree types when separately considering all trees and coniferous trees. However, few generalists such as *P. adscendens* were independent of their substrate, indicating that overgrowing and displacing other lichens is its main life strategy. The colonization of lichenized fungi on leaves of broadleaved trees seems to be rather independent of the tree species as we found no relationship between the two. Leaves and needles of different tree species differed in various traits including nutrition, persistence, specific leaf area, shapes, pH, and water content. However, there is no solid evidence that lichens can uptake nutrients from host leaves and needles. Foliicolous lichens usually grow onto evergreen needles as they develop thalli with reproductive organs and complete their life cycle (Lücking and Bernecker-Lücking, 2002). However, in our study, we were also able to detect lichenized fungi on deciduous tree species, including *Larix decidua* as well as broadleaved trees. We also detect algae and cyanobacteria in leaves and needles across the 12 temperate tree species. Our study is considered among the first to demonstrate the specificity of lichenized fungi on different host leaves/needles.

## Conclusion

The monitoring of lichens is generally based on fruiting body surveys. Overlooked or even invisible lichens are thus usually neglected. Here, we demonstrated that Next-Generation Sequencing can be used to augment the lichen monitoring, which adds significantly to previous knowledge. Conifers showed a higher lichenized fungal richness compared to broadleaved trees. Moreover, the lichenized fungal generalist, *P. adscendens* was the most successful lichenized fungi dominating the majority of leaves and needles of the temperate tree species. We conclude that conifers are important for maintaining the biodiversity of foliicolous lichens. Future studies should investigate the interactions among lichenized fungi and environmental conditions that favor the conservation of lichens.

## Data availability statement

The datasets presented in this study can be found in online repositories. The names of the repository/repositories and accession number(s) can be found at: https://www.ncbi.nlm.nih.gov/, PRJNA753096.

## Author contributions

WP and E-DS conceived and designed the study. BT, WP, SS, SH, A-SL, and SW collected the samples and metadata. WP and FB contributed reagents and laboratory equipment. BT, WP, and SW led the DNA analysis. SS and GG led the water content and pH measurement. SW led bioinformatics. BT, LJ, and WP led the microbial taxonomy and data analyses. HG performed the fruiting body survey. BT, SS, MN, and WP wrote the manuscript. MN and WP supervised BT. SS, MN, E-DS, and FB reviewed and gave comments and suggestions for the manuscript. All authors gave final approval for manuscript submission.

## Funding

This work has been partially funded by the internal research budget of WP to the Department of Soil Ecology, UFZ-Helmholtz Centre for Environmental Research. LJ appreciates the financial support from the China Scholarship Council (No. 201906600038).

## Conflict of interest

The authors declare that the research was conducted in the absence of any commercial or financial relationships that could be construed as a potential conflict of interest.

## Publisher's note

All claims expressed in this article are solely those of the authors and do not necessarily represent those of their affiliated organizations, or those of the publisher, the editors and the reviewers. Any product that may be evaluated in this article, or claim that may be made by its manufacturer, is not guaranteed or endorsed by the publisher.
