# Supplementary material for: More than you can see: Unraveling the ecology and biodiversity of lichenized fungi associated with leaves and needles of 12 temperate tree species using high-throughput sequencing
Source: Front Microbiol. 2022 Sep 16;13:907531. doi: 10.3389/fmicb.2022.907531 (PMC9523249; doi:10.3389/fmicb.2022.907531)

Supplementary Material

More than you can see: Unraveling the ecology and biodiversity of lichenized fungi associated with leaves and needles of 12 temperate tree species using high throughput sequencing.

Benjawan Tanunchai[1,2¶], Simon Andreas Schroeter [3¶], Li Ji[1,4], Sara Fareed Mohamed Wahdan[1,5], Shakhawat Hossen[1,6], Ann-Sophie Lehnert[3], Hagen Grünberg[7], Gerd Gleixner[3], François Buscot[1,8], Ernst-Detlef Schulze[3], Matthias Noll[2,6*], Witoon Purahong[1*]

[1] UFZ-Helmholtz Centre for Environmental Research, Department of Soil Ecology, Theodor-Lieser-Str. 4, 06120 Halle (Saale), Germany.

[2] Bayreuth Center of Ecology and Environmental Research (BayCEER), University of Bayreuth, Bayreuth, Germany

[3] Max Planck Institute for Biogeochemistry, Biogeochemical Processes Department, Hans-Knöll-Str. 10, 07745 Jena, Germany.

[4] School of Forestry, Central South of Forestry and Technology, 410004, Changsha, P.R. China.

[5] Department of Botany and Microbiology, Faculty of Science, Suez Canal University, 41522 Ismailia, Egypt.

[6] Institute of Bioanalysis, Coburg University of Applied Sciences and Arts, Coburg, Germany.

[7] Preßwitzer Straße 13, 07333 Unterwellenborn, Germany, Lichen-Expert Thüringen.

[8] German Centre for Integrative Biodiversity Research (iDiv) Halle-Jena-Leipzig, Deutscher Platz 5e, 04103 Leipzig, Germany.

¶These authors contribute equally to this work.

***Correspondence:** Corresponding Authors: Witoon Purahong (witoon.purahong@ufz.de; Tel.: 0345-5585207); Matthias Noll (Matthias.noll@hs-coburg.de; Tel.: 09561-8530833)

Keywords: Illumina MiSeq, ITS2, *Physcia adscendens*, red-list lichenized fungi, foliicolous lichens.

Table S1. Comparisons between lichenized fungal community compositions associated with all tree species (i), broadleaved (ii) and coniferous tree species (iii) using analysis of similarities (ANOSIM) and non-parametric multivariate analysis of variance (NPMANOVA) based on relative abundance data and the Bray-Curtis distance measure. Bold letters indicate statistical significance.

(i) All tree species

Comparison	ANOSIM	NPMANOVA
Q. robur vs. *F. excelsior*	$R = 0.00$, $P = 1.000$	Pseudo$F = 0.64$, $P = 0.594$
Q. robur vs. *P. avium*	$R = 0.00$, $P = 0.397$	Pseudo$F = 1.11$, $P = 0.347$
Q. robur vs. *P. menziesii*	$R = 0.02$, $P = 0.317$	Pseudo$F = 1.20$, $P = 0.314$
Q. robur vs. *P. abies*	$R = 0.32$, **$P = 0.046$**	Pseudo$F = 2.50$, $P = 0.064$
Q. robur vs. *P. sylvestris*	$R = 0.72$, **$P = 0.008$**	Pseudo$F = 4.54$, **$P = 0.009$**
Q. robur vs. *L. decidua*	$R = 0.64$, **$P = 0.007$**	Pseudo$F = 6.07$, **$P = 0.008$**
F. excelsior vs. *P. avium*	$R = 0.00$, $P = 0.571$	Pseudo$F = 1.11$, $P = 0.435$
F. excelsior vs. *P. menziesii*	$R = 0.00$, $P = 0.386$	Pseudo$F = 1.52$, $P = 0.218$
F. excelsior vs. *P. abies*	$R = 0.10$, $P = 0.214$	Pseudo$F = 2.22$, $P = 0.153$
F. excelsior vs. *P. sylvestris*	$R = 0.34$, **$P = 0.016$**	Pseudo$F = 3.18$, **$P = 0.009$**

Comparison	ANOSIM	NPMANOVA
F. excelsior vs. *L. decidua*	$R = 0.34$, **$P = 0.034$**	Pseudo$F = 5.45$, **$P = 0.024$**
P. avium vs. *P. menziesii*	$R = 0.00$, $P = 0.378$	Pseudo$F = 0.88$, $P = 0.514$
P. avium vs. *P. abies*	$R = 0.12$, $P = 0.233$	Pseudo$F = 0.87$, $P = 0.485$
P. avium vs. *P. sylvestris*	$R = 0.77$, $P = 0.018$	Pseudo$F = 4.45$, **$P = 0.018$**
P. avium vs. *L. decidua*	$R = 0.31$, $P = 0.104$	Pseudo$F = 1.84$, $P = 0.109$
P. menziesii vs. *P. abies*	$R = 0.12$, $P = 0.181$	Pseudo$F = 1.54$, $P = 0.184$
P. menziesii vs. *P. sylvestris*	$R = 0.76$, **$P = 0.008$**	Pseudo$F = 4.79$, **$P = 0.008$**
P. menziesii vs. *L. decidua*	$R = 0.31$, **$P = 0.048$**	Pseudo$F = 3.54$, **$P = 0.033$**
P. abies vs. *P. sylvestris*	$R = 0.79$, **$P = 0.009$**	Pseudo$F = 6.53$, **$P = 0.009$**
P. abies vs. *L. decidua*	$R = 0.13$, $P = 0.078$	Pseudo$F = 1.76$, **$P = 0.042$**
P. sylvestris vs. *L. decidua*	$R = 0.97$, **$P = 0.007$**	Pseudo$F = 19.28$, **$P = 0.008$**

(ii) Broadleaved tree species

Comparison	ANOSIM	NPMANOVA
Q. robur vs. *F. excelsior*	$R = 0.00$, $P = 1.000$	Pseudo$F = 0.64$, $P = 0.599$
Q. robur vs. *P. avium*	$R = 0.00$, $P = 0.403$	Pseudo$F = 1.11$, $P = 0.342$
F. excelsior vs. *P. avium*	$R = 0.00$, $P = 0.574$	Pseudo$F = 1.11$, $P = 0.425$

(iii) Coniferous tree species

Comparison	ANOSIM	NPMANOVA
P. menziesii vs. *P. abies*	$R = 0.12$,<br>$P = 0.181$	Pseudo$F = 1.54$,<br>$P = 0.180$
P. menziesii vs. *P. sylvestris*	$R = 0.76$,<br>$\boldsymbol{P = 0.008}$	Pseudo$F = 4.79$,<br>$\boldsymbol{P = 0.009}$
P. menziesii vs. *L. decidua*	$R = 0.31$,<br>$\boldsymbol{P = 0.048}$	Pseudo$F = 3.54$,<br>$\boldsymbol{P = 0.033}$
P. abies vs. *P. sylvestris*	$R = 0.79$,<br>$\boldsymbol{P = 0.008}$	Pseudo$F = 6.53$,<br>$\boldsymbol{P = 0.008}$
P. abies vs. *L. decidua*	$R = 0.13$,<br>$P = 0.078$	Pseudo$F = 1.76$,<br>$\boldsymbol{P = 0.038}$
P. sylvestris vs. *L. decidua*	$R = 0.97$,<br>$\boldsymbol{P = 0.007}$	Pseudo$F = 19.28$,<br>$\boldsymbol{P = 0.007}$

Figure S1. The 12 temperate tree species located in the Hainich-Dün region of Thuringia, Germany (51°12'N 10°18'E).

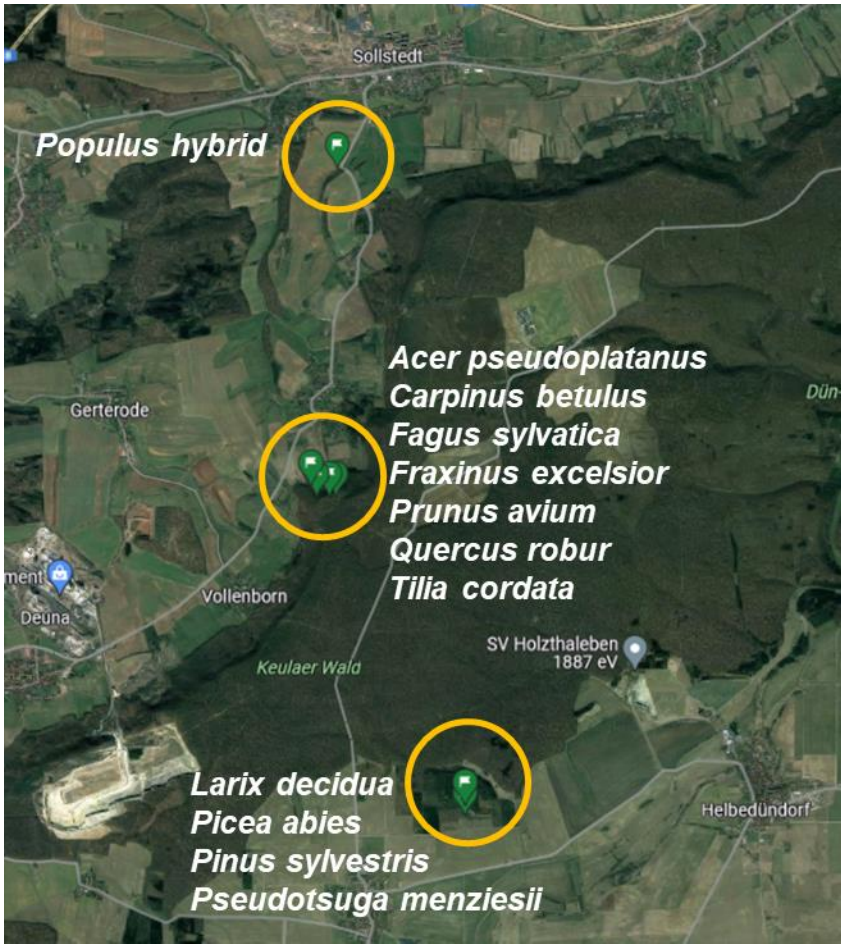

Google Maps, 2022. Hainich-Dün region of Thuringia. Google Maps [online] Available at: https://www.google.com/maps, [Accessed 12 July 2022].

Figure S2. Principal coordinates showing lichenized fungal community compositions in broadleaved tree species. Effects of tree species (only tree species with more than three replicates being considered) were tested with one-way PERMANOVA (based on relative abundance data and the Bray-Curtis distance measure). Color code of each data point indicates leaves and needles of different tree species and is consistent with the color code of Figure 2.

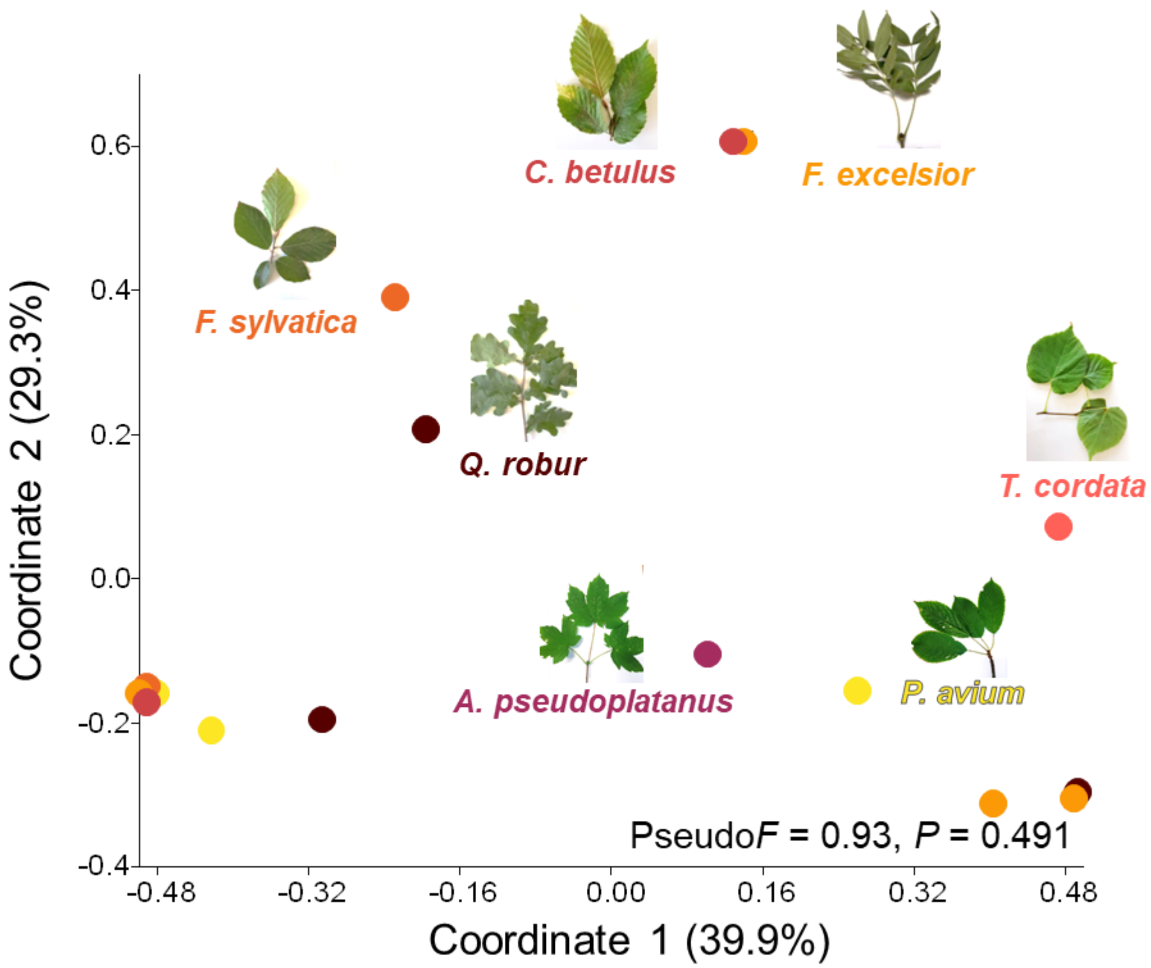

Figure S3 Leaf/needle water content and pH of the 12 temperate tree species. Color code of each data point indicates leaves and needles of different tree species and is consistent with the color code of Figure 2. Yellow-red-brown color tone refers to the broadleaved tree species and blue-green color tone refers to the coniferous tree species. The statistical differences ($P < 0.05$) as indicated by letters among different tree species were performed using one-way ANOVA or Kruskal-Wallis test.

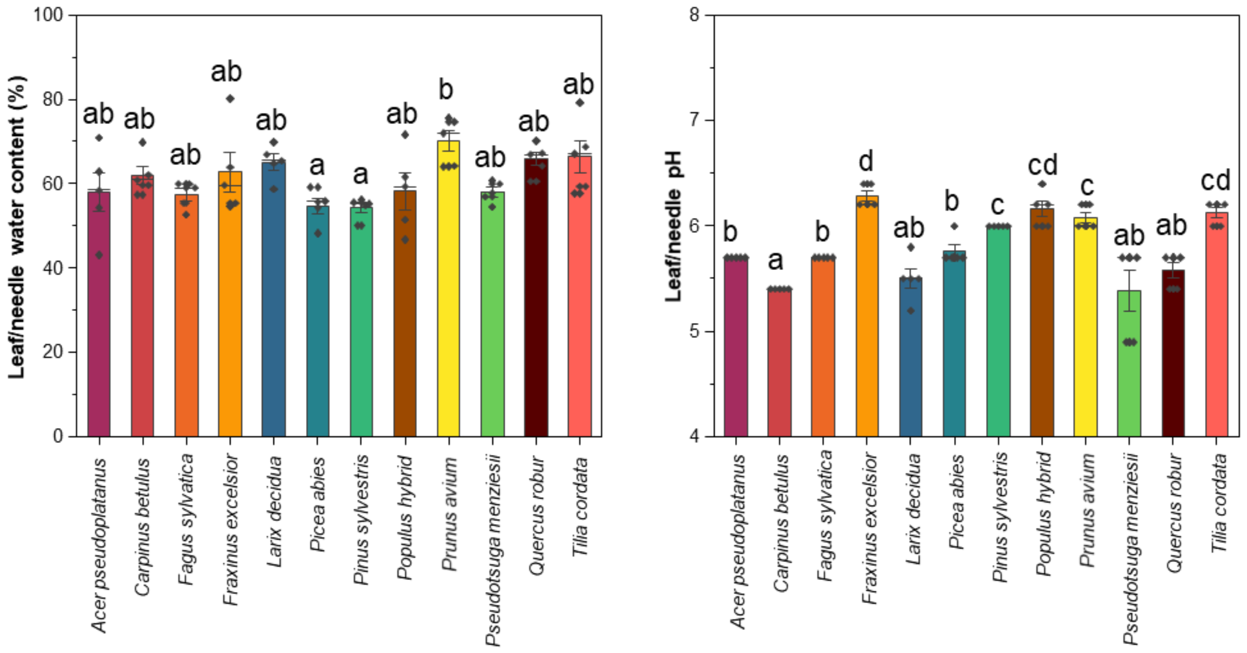

Supplement: Supplementary file 1 [file Data_Sheet_1.pdf]
